# The data on exploratory factor analysis of factors influencing employees effectiveness for responding to crisis in Iran military hospitals

**DOI:** 10.1016/j.dib.2018.05.117

**Published:** 2018-05-24

**Authors:** Ahmad Amerioun, Abouzar Alidadi, Rohollah Zaboli, Mojtaba Sepandi

**Affiliations:** a Health Services Management, Health Management Research Center, Baqiyatallah University of Medical Sciences, Tehran, Iran; b Health Services Management, Baghiatallah University of Medical Sciences, Tehran, Iran; c Health Services Management, Faculty of Health, Baghiatallah University of Medical Sciences, Tehran, Iran; d Department of Epidemiology and Biostatistics, Health Faculty, Baqiyatallah University of Medical Sciences, Tehran, Iran

**Keywords:** Employees effectiveness, Crisis response, Effective response to crisis factor questionnaire, Iran

## Abstract

The article presents the data on the exploratory analysis of factors involved in employees’ effectiveness for responding to crisis in Iran׳s military hospitals. This research was a descriptive exploratory study. The statistical population included the 561 medical and nonmedical staff of three military hospitals. Two researcher-made questionnaires were used to collect data, and reliability and validity of the questionnaires were confirmed. The exploratory factor analysis (EFA) method was used to classify, clarify, and explain study factors and the infrastructural structure. At the end, 473 questionnaires were found appropriate for the final analysis. Based on results of the exploratory factor analysis (EFA), 8 criteria were identified as the main factors involved in employees’ effectiveness for responding to crisis. According to Friedman test results, organizational factors were the most important factors influencing employees’ effectiveness with a mean score of 3.76 of 5. Responding to crisis was the most important variable factor involved response to crisis with a mean score of 3.74 of 5.

**Specifications Table**

**Table:** 

Subject area	Health
More specific subject area	Health management
Type of data	Tables and Figures
How data was acquired	Two researcher-made questionnaires were used to collect data from the medical and nonmedical staff of three military hospitals. The reliability and validity of the questionnaires were confirmed.
Data format	Analyzed
Experimental factors	The questionnaire was prepared by fusing several standard questionnaires and notions, questions, and statements raised by crisis professors and experts.
Experimental features	The exploratory factor analysis (EFA) method was used to classify, clarify, and explain study factors and the infrastructural structure.
Data source location	Tehran, Tehran province, Iran.
Data accessibility	Data are included in this article

**Value of the data**

• For success and effectiveness of medical and nonmedical measures in hospitals in response to crisis, many factors such as facilities and expert human force is necessary to be prepared. Employees’ performance is assessed base on following two substantial concepts: effectiveness and efficiency [1], [2], [3], [4], [5], [6].
• This data include the exploratory analysis on factors involved in employees’ effectiveness for responding to crisis in Iran׳s military hospitals.
• The data in this article indicates that there are 8 criteria as the main factors involved in employees’ effectiveness for responding to crisis.
• The analyzed data in this article shows that organizational factors are the most important factors for effectiveness of employees during crisis.
• The data included in this research are expected to be utilized more effectively in future studies to collect data on factors influencing effectiveness of employees for responding to crisis in other organizations.

## 1 Data

Analysis of demographic properties of the study population indicated that 263 (55.6%) participants in this study had taken crisis management courses, 330 (69.8%) had attended crisis management programs, and 91 (19.2%) had responsibilities in crisis programs. Therefore, the minimum inclusion criterion was met. The factor analysis of employees’ effectiveness with 38 statements, which was carried out by obtaining the main components in accord with Table 1 based on the eigenvalue column, factors with eigenvalues higher than one led to identification of four factors. Each factor׳s share of variance of the 38 statements is shown in the variance percent column. The first factor had the largest share (46.670 with an eigenvalue of 17.735) of variance, whereas the fourth factor had the smallest share (2.925 with an eigenvalue of 1.112) of variance of 38 statements. In sum, all of the four factors with eigenvalues larger than one explained 57.577% of variance of 38 statements. Since the eigenvalues of these factors were larger than one and factor loading of each statement was close to one, the factorial validity of statements related to employees’ effectiveness is satisfactory by accepting the related hypotheses.

**Table 1:** Eigenvalues, variance percentage, and cumulative variance of factors identified after a varimax rotation.

**No.**	**Questionnaire dimensions**	**Factor**	**Rotation sums of squared loadings**		
			**Eigenvalue**	**Variance (%)**	**Cumulative variance (%)**
1	Employees effectiveness factors	Personal factors	17.735	46.670	46.670
2		Organizational factors	1.680	4.422	51.092
3		Group factors	1.353	3.560	54.651
4		Administrative factors	1.112	2.925	57.577
5	Response to crisis	Responding to crisis	3.735	28.730	28.730
6		Resource supply	2.550	19.614	48.345
7		Capacity and potential for responding to crisis	1.546	11.893	60.237
8		Crisis response expert workgroup	1.075	8.272	68.510

As the crisis response data in Table 1 indicate the first factor had the largest share (3.735 with an eigenvalue of 28.730) of variance of 13 statements, whereas the fourth factor had the smallest share (1.075 with an eigenvalue of 8.272). In summary, all 4 factors with eigenvalues higher than one explained 68.509 of variance of 13 statements. Therefore, it is concluded that factorial validity of statements related to crisis response variable is satisfactory by accepting the hypotheses.

**Fig. 1:**
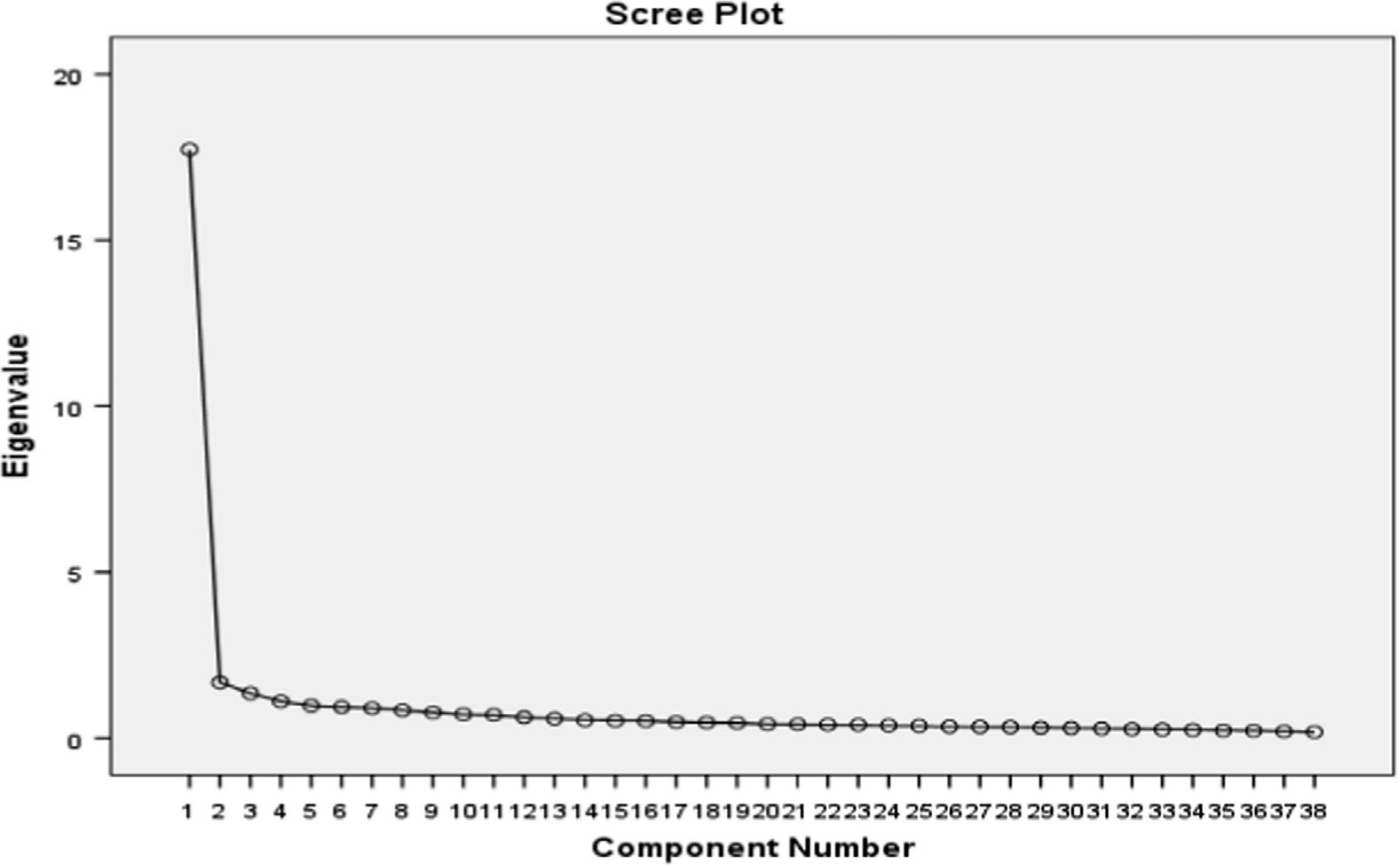
Cattell׳s screen plot of 4 components of employees’ effectiveness.

**Fig. 2:**
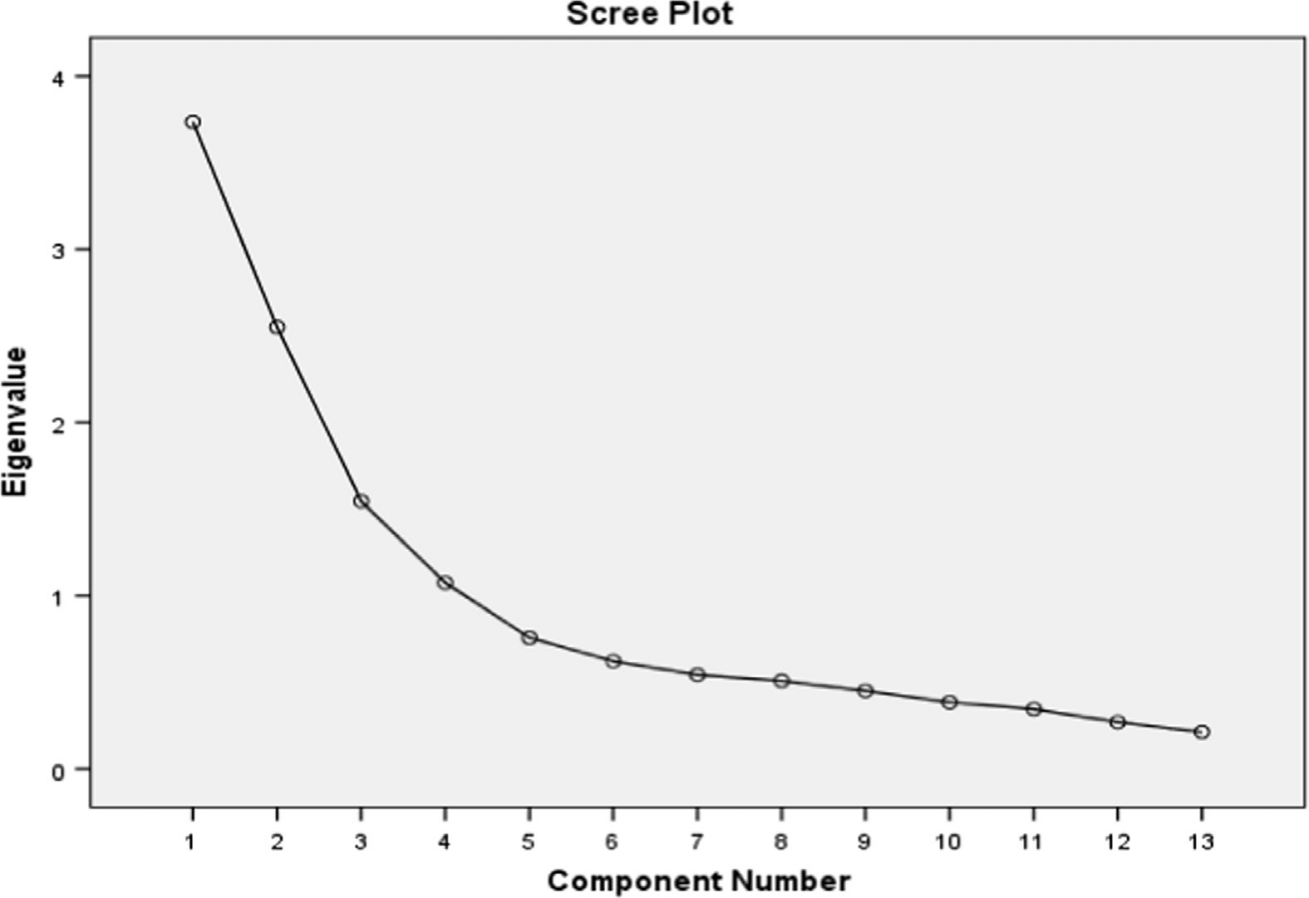
Cattell׳s screen plot of 4 components of crisis response.

The screen plots presented for both variables in the following visually illustrate results of the table of variance explained by factors of both variables based on suitable number of factors. That is to say, similar to eigenvalue, this plot helps determine the number of factors. According to Fig. 1, Fig. 2 in the case of both variables, eigenvalues of 4 factors are higher than one. In other words, the 13 crisis response statements and 38 employees’ effectiveness statements can be reduced to four factors separately.

Results of analysis of correlations between factors of the employees’ effectiveness and crisis response variables indicated that correlation coefficient of all factors was close to zero, which reflect their lack of correlation. Hence, since all factors of the employees’ effectiveness and crisis response variables were uncorrelated, orthogonal varimax rotation methods were used to rotate factors.

According to Table 1, factors influencing effectiveness of employees for responding to crisis were summarized into eight factors using the Principle Component Analysis (PCA) and varimax rotation methods. Finally, the eight factors were named with the aid of the research steering committee. The factors and components of each factor are introduced in the following. Research findings showed that the following eight factors were identified and prioritized as factors influencing employees’ effectiveness in responding to crisis: responding to crisis, resource supply, responding capacity and ability, expert workgroup, personal factors, group factors, organizational factors, and administrative factors.

Results of the Kaiser-Meyer-Olkin (KMO = 0.973) and Bartlett׳s test at significance level of <0.01 (sig = 0.001 is rejected) for employees’ effectiveness are show in Table 2. These results suggest that factor analysis was suitable for these statements. In all statements except for questions q4 and q8 the factor loading is higher than 0.5 which indicates that these statements can optimally explain corresponding variances and the questions are significance. Hence, by omitting questions q4 and q8 these statements become suitable for determining effectiveness factors in this research.

**Table 2:** Factor analysis, KMO, and Bartlett׳s tests for each research variable as regards employees’ effectiveness.

**Factor titles**	**Questions**	**Statements**	**Factor loading**	**KMO**	**BT**	**DF**	***p*-Value**
Administrative factors	q1	Training resources management and organization based on standards and employees needs assessments	0.77	0.910	1816.92	21	0.001
	q2	Time management in changing use of employees and workplace from normal to critical mode	0.80				
	q3	Senior managers’ knowledge of employees’ substantial capabilities and duties	0.72				
	q4	Suitability of managers’ management method with employees status and competencies	0.70				
	q5	Employing staff in proportion to different situations in different types of crisis	0.76				
	q6	Speed of operational plans based on urgent action scenario	0.37				
	q7	Selection of employees based on professional characteristics and qualification	0.72				
	q8	Organizing a transportation system for transferring victims from the crisis scene to hospital	0.32				
Personal factors	q9	Employees’ personal ability to cooperate with other medical teams during crisis	0.70	0.949	3016.12	55	0.001
	q10	Personal mobility and movement of employees during crisis	0.78				
	q11	Quality and type of equipment used for time of crisis	0.78				
	q12	Employees’ knowledge of nature and types of crises	0.81				
	q13	Employees’ knowledge of available facilities and resources during crisis	0.74				
	q14	Employee’s skills for accomplishing tasks properly during crisis	0.72				
	q15	Proportionality of the assigned task or mental/stressful condition of workplace to employees	0.73				
	q16	Employees’ motivation and interest in cooperating with training programs	0.69				
	q17	Employees independence in accomplishing tasks during crisis	0.72				
	q18	Paying attention to opinions, suggestions, and complaints of employees for improving activities effectiveness	0.74				
	q19	Elimination of negative feeling of inequality and injustice in workplace to prevent under-activity	0.76				
Group factors	q20	Coordination, sharing of efforts, and teamwork	0.78	0.884	1518.71	28	0.001
	q21	Defining group activities for employees	0.75				
	q22	Universal and active cooperation of employees in determining organization’s goals and decisions	0.75				
	q23	Improving jihad spirit in medical and nursing staff	0.76				
	q24	Proper organization of major and alternate professional teams for coping with crisis	0.79				
	q25	Dominance of spontaneous and voluntary actions by employees in provision of services	0.79				
	q26	Interaction, sharing efforts, correlation, and group coherence among employees	0.75				
Organizational factors	q27	Creating mutual trust between managers and staff	0.76	0.939	2994.88	66	0.001
	q28	Improvement of human relations in workplace and emotional commitment	0.69				
	q29	Flexibility and improvement of operational methods, facilities, and equipment	0.62				
	q30	Aligning employee goals with organization’s goals	0.72				
	q31	Holding training courses and workshops matching staff characteristics	0.72				
	q32	Support for employees welfare, reward system, and satisfactory promotions	0.71				
	q33	System of suitable, actual, and effective performance assessment	0.65				
	q34	Performance assessment for identifying weaknesses and strengths	0.77				
	q35	Increasing motivation and accountability of employees	0.71				
	q36	Proportionality of employees place and skills during crisis	0.69				
	q37	Deployment and organization of a system of managing unexpected hospital accidents	0.74				
	q38	professions and workers for professional promotion an movement of employees	0.72				
Sum of KMO and Bartlett’s questions				0.973	12014.87	703	0.001

In addition, results of the KMO (=0.956) and Bartlett׳s tests at significance level of <0.01 (sig = 0.001 is rejected) for crisis response in Table 3 indicate that factor analysis is suitable for these statements. In all statements, the factor larger than 0.05 suggests that the statements can optimally explain variances of their related factors, and thus the questions are significant.

**Table 3:** Factor analysis, KMO, and Bartlett׳s tests for each research variable as regards response to crisis.

**Factor titles**	**Questions**	**Statements**	**Factor loading**	**KMO**	**BT**	**DF**	***p*-Value**
Responding to crisis	q39	Availability of a predetermined standard response procedure	0.82	0.804	586.2	6	0.001
	q40	Availability of a response program based on clear specific descriptions of duties	0.84				
	q41	Availability of a response plan supervised by a single commander and specified members	0.79				
	q42	Emphasizing responsibility with supervision and control of consumables and constructional expenses	0.78				
Resource supply	q43	Support of relief and service organizations in response to disasters	0.73	0.500	62.49	1	0.001
	q44	Ease of access to emergency teams for all employees	0.81				
Responding capacity	q45	A changeable response program structure based on type of accident	0.77	0.754	451.01	6	0.001
	q46	Coverage of response program in hospital by hospital staff	0.76				
	q47	Existence of flexible and diverse procedures on different crisis response levels in hospitals	0.85				
	q48	Existence of stress management programs for employees working under critical conditions	.810				
Expert workgroups	q49	Existence of expert work groups for crisis response	0.72	0.655	167.82	3	0.001
	q50	Taking professional adequate training courses on crisis response	0.80				
	q51	Training hours in hospital crisis management programs	0.84				
Sum of KMO and Bartlett questions				0.956	4033.148	78	0.001

Results in Table 4 indicate that according to respondents, among the factors influencing employees’ effectiveness, organizational factors are the most important with a mean score of 3.76 of 5, whereas administrative factors are the least important with a mean score of 1.09 of 5. Among the crisis response criteria, the responding process has the highest level of importance with a mean score of 3.47 of 5, while mobilization and supply of resources has the lowest importance with a mean score of 1.06 of 5. In addition, other factors are shown in the aforementioned table in the order of significance.

**Table 4:** Ranking of factors influencing employees’ effectiveness for responding to crisis.

**Questionnaire dimensions**	**No.**	**Components**	**Priority**	**Mean of 5**
Employees effectiveness factors	1	Personal factors	Second	3.18
	2	Organizational factors	First	3.76
	3	Group factors	Fourth	1.09
	4	Administrative factors	Third	1.96
Response to crisis	5	Responding to crisis	First	3.47
	6	Resource supply	Fourth	1.06
	7	Capacity and potential for responding to crisis	Second	3.45
	8	Expert work groups	Third	2.02

## 2 Experimental design, materials and methods

This research is an exploratory study that was conducted using the field research method. The study population included all of the medical and nonmedical staff of three military hospitals in Tehran City. Samples were collected using the stratified random sampling method from all of the in-patient, out-patient, administrative, engineering, and other wards of three military hospitals. Data was collected using the employees effectiveness and crisis response researcher-made questionnaires, which were prepared by fusing several standard questionnaires and notions, questions, and statements raised by crisis professors and experts. With a sample loss of 10% a total of 561 samples were included in the research23. Questionnaires validities were calculated for all questions to be higher than 0.89 and 0.92 based on opinions of 8 experts using the Lawshe (1986) CVI and CVR forms, and reliability of the questionnaires was higher than 0.7 using the Cronbach׳s alpha of both questionnaires. The inclusion criterion was reference to presence in one course or program or responsibilities in the past or present crisis management records. The finalized questionnaire was distributed among the samples, and finally 473 appropriate 473 were analyzed after the pre-processing especially omission of indifferent samples. Afterwards, through exploratory factor analysis the factors were categorized and descriptive statistical methods (including mean and standard deviation) were used to analyze the findings. Friedman test was also used to rank the indices, and examinations of skewness and kurtosis were used to determine normality of variables. Calculations were carried out in SPSS version 20 at a significance level of *P* < 0.05.
