# Supplementary material for: The data on exploratory factor analysis of factors influencing employees effectiveness for responding to crisis in Iran military hospitals
Source: Data Brief. 2018 May 24;19:1522–9. doi: 10.1016/j.dib.2018.05.117 (PMC6140286; doi:10.1016/j.dib.2018.05.117)
Supplement: Supplementary file 1 — Supplementary material. [file mmc1.doc]

Dear editor(s) of Data in Brief

We would be grateful if you would consider the attached manuscript “**The data on exploratory factor analysis of factors influencing employees effectiveness for responding to crisis in Iran military hospitals”** for publication in valuable Journal Data in Brief. We declare that this manuscript, or part of it, has neither been published nor is currently under consideration for publication by any other journal. The data presented in this article is new original data. We also declare that we have read the manuscript and approved submission to Journal of Data in Brief. None of the authors of this paper have any conflicts of interest to declare.

Best Regards,

Abouzar Alidadi

*Health Services Management, Baghiatallah University of Medical Sciences, Tehran, Iran*

E-mail address: [Aboozaralidadi3@gmail.com](mailto:Aboozaralidadi3@gmail.com)
